# Hormone receptor status, tumor characteristics, and prognosis: a prospective cohort of breast cancer patients

**DOI:** 10.1186/bcr1639

**Published:** 2007-01-19

**Authors:** Lisa K Dunnwald, Mary Anne Rossing, Christopher I Li

**Affiliations:** 1 Department of Epidemiology, University of Washington, Box 356113, 1959 NE Pacific St., Seattle, WA 98195, USA; 2 Division of Public Health Sciences, Fred Hutchinson Cancer Research Center, 1100 Fairview Avenue N., Seattle, WA 98109-1024, USA

## Abstract

**Background:**

Breast cancer patients with tumors that are estrogen receptor (ER)-positive and/or progesterone receptor (PR)-positive have lower risks of mortality after their diagnosis compared to women with ER- and/or PR-negative disease. However, few studies have evaluated variations in the risks of breast cancer-specific mortality across ER/PR status by either demographic or clinical characteristics.

**Methods:**

Using data from 11 population-based cancer registries that participate in the SEER (Surveillance, Epidemiology, and End Results) program, 155,175 women at least 30 years old with a primary diagnosis of invasive breast carcinoma from 1990 to 2001 were included in the study. Associations between joint hormone receptor status and breast cancer mortality risk within categories of diagnosis age, diagnosis year, race/ethnicity, histologic tumor type, stage, grade, size, and axillary lymph node status were evaluated using the Cox proportional hazards model.

**Results:**

Compared to ER+/PR+ cases, elevations in risk of mortality were observed across all subcategories of age at diagnosis, ranging from 1.2- to 1.5-fold differences for ER+/PR- cases, 1.5- to 2.1-fold differences for ER-/PR+ cases, and 2.1- to 2.6-fold differences for ER-/PR- cases. Greater differences were observed in analyses stratified by grade; among women with low-grade lesions, ER-/PR- patients had a 2.6-fold (95% confidence interval [CI] 1.7 to 3.9) to 3.1-fold (95% CI 2.8 to 3.4) increased risk of mortality compared to ER+/PR+ patients, but among women with high-grade lesions, they had a 2.1-fold (95% CI 1.9 to 2.2) to 2.3-fold (95% CI 1.8 to 2.8) increased risk.

**Conclusion:**

Compared to women with ER+/PR+ tumors, women with ER+/PR-, ER-/PR+, or ER-/PR- tumors experienced higher risks of mortality, which were largely independent of the various demographic and clinical tumor characteristics assessed in this study. The higher relative mortality risks identified among ER-/PR- patients with small or low-grade tumors raise the question of whether there may be a beneficial role for adjuvant chemotherapy in this population.

## Introduction

Breast cancer patients with tumors that are estrogen receptor (ER)-positive and/or progesterone receptor (PR)-positive have lower risks of mortality after their diagnosis compared to women with ER- and/or PR-negative disease [1-6]. Clinical trials have also shown that the survival advantage for women with hormone receptor-positive tumors is enhanced by treatment with adjuvant hormonal and/or chemotherapeutic regimens [7-9]. However, few studies have evaluated variations in the risks of breast cancer-specific mortality across ER/PR status by either demographic or clinical characteristics. The goal of this study was to determine whether the greater relative risk of breast cancer mortality observed among women with hormone receptor-negative tumors was similar for those with different demographic characteristics, such as race/ethnicity, age of cancer diagnosis, and year of cancer diagnosis, and for those with different tumor characteristics, such as stage, grade, tumor size, and histology, using data from Surveillance, Epidemiology, and End Results (SEER), the population-based tumor registry program of the National Cancer Institute (NCI). Such an assessment may further elucidate the relationships between various prognostic indicators in breast cancer and provide knowledge regarding the prognostic utility of ER/PR status by demographic and clinical tumor characteristics.

## Materials and methods

### Patient selection

The cohort for this study was assembled using data from the NCI's SEER program in the United States [10]. Since the early 1970s, the SEER program has collected incidence and survival data from population-based cancer registries spanning five states and four metropolitan areas: Connecticut, Hawaii, Iowa, New Mexico, Utah, San Francisco-Oakland (CA), Detroit (MI), Seattle (Puget Sound) (WA), and Atlanta (GA). In 1992, two population-based registries were added: San Jose-Monterey (CA) and Los Angeles (CA). SEER began collecting data on ER and PR status in 1990 for breast cancer cases. Therefore, only data from 1 January 1990 through 31 December 2001 were included in this analysis.

We identified 209,276 women at least 30 years old with a primary diagnosis of invasive carcinoma of the breast as potentially eligible subjects for this study. Women under the age of 30 years were excluded because the occurrence of breast cancer among this age group is rare. Also excluded were 1,873 women whose breast carcinomas were diagnosed only by autopsy or death certificate or whose diagnostic confirmation of breast cancer was unknown. Because our primary analysis focused on associations between joint ER/PR status and breast cancer-specific mortality, we excluded 50,291 women missing tumor marker data for ER and/or PR (24% of the total potentially eligible subjects). After the noted exclusions, 155,175 subjects were included in the study.

Women were categorized into four groups according to their joint ER/PR status: ER+/PR+, ER+/PR-, ER-/PR+, and ER-/PR-. The SEER registries provide information on age of diagnosis, year of diagnosis, and race/ethnicity. Data on other clinical characteristics, such as tumor histology, grade, stage (American Joint Committee on Cancer [AJCC] classification system), and size, were also available. Additionally, SEER collects data regarding the first course of cancer-directed surgical and radiation treatment interventions. However, SEER does not make data on adjuvant chemotherapy or hormonal therapy publicly available. SEER does not collect information on other relevant factors such as reproductive history, anthropometrics, medical history, family history of cancer, or cancer screening history.

### Outcome measures

Each SEER registry routinely updates vital status and follow-up information for all patients with cancer. Survival time is calculated in months by using the subject's date of breast cancer diagnosis and one of the following: (a) date of death, (b) date last known to be alive, or (c) the most recent follow-up cutoff date. The follow-up cutoff date for the SEER data used in our analysis was 31 December 2001. Because we were interested primarily in cause-specific mortality, our outcome of interest was death due to breast cancer as indicated by cause-of-death International Classification of Diseases codes. Women who died of causes other than breast cancer were censored at their date of death.

### Statistical analysis

The statistical software program Stata for Macintosh version 9.1 (StataCorp LP, College Station, TX, USA) was used to perform all analyses. Women with ER+/PR+ tumors served as the referent category in all analyses because this was the largest ER/PR subgroup. Associations between joint ER/PR receptor status and breast cancer mortality risk within categories of diagnosis age (<50, 50 to 64, ≥65 years), diagnosis year (3-year intervals: 1990 to 1992, 1993 to 1995, 1996 to 1998, and 1999 to 2001), race/ethnicity (non-Hispanic white, terminology used is base on the SEER registries descriptions. If you would prefer to change, OK. African-American, Native American, Asian/Pacific Islander, Hispanic white), histologic tumor type (ductal, lobular, ductal/lobular, inflammatory, mucinous, tubular, comedo, medullary, papillary), AJCC stage grouping (I, II, III, IV), SEER grade (I, well differentiated; II, moderately differentiated; III, poorly differentiated; IV, undifferentiated), size (0 to 1.9, 2 to 5, >5 cm), and axillary lymph node status (binary – yes/no or 0, 1 to 3, 4 to 10, >11 positive) were estimated using the Cox proportional hazards model. Unknown factors or those with missing data were excluded from all model estimates. We used likelihood ratio testing to evaluate whether variations in mortality risks by these categories were statistically significant. Hazard ratios (HRs) and their associated 95% confidence intervals (CIs) were calculated as estimates of relative risks of mortality [11,12]. Based on log-log survival curves, proportional hazards assumption in these data was validated for all categories of ER/PR status except for ER-PR+. This is likely a consequence of ER-/PR+ being the rarest ER/PR subtype, and for this subgroup, a gross violation of the proportional hazards assumption was not observed. In our analyses, we evaluated age of diagnosis, year of diagnosis, SEER registry site (stratified variable), race/ethnicity, histologic type, tumor size, stage, grade, and lymph node status as potential confounders in multivariate Cox regression modeling. We also evaluated the possible influence of initial cancer treatments (surgery/radiation – yes/no) given that treatment recommendations are based on tumor characteristics and that therapeutic interventions are associated with survival. We assessed whether associations between ER/PR status and demographic/clinical characteristics were different for varying levels of a factor by fitting interaction models. For each model, dummy variables were created and each represented the combination of an ER/PR category and the characteristic assessed, with ER+PR+ and the following characteristics as referent categories: age less than 50 years, year of diagnosis 1990 to 1992, non-Hispanic white, tumor grade 1, tumor stage 1, tumor size less than 2 cm, negative lymph nodes, and ductal tumor histology. Each full model with the cross-categorized variables was compared to the reduced model (main effects only) by likelihood ratio testing to assess statistical significance. We also estimated trends in HRs for each ER/PR profile and the following: (a) age at diagnosis, per 5 years (30 to <35 years referent), (b) diagnosis year, per year (1990 referent), (c) tumor stage (stage 1 referent), (d) tumor size (0 to <1 cm [referent], 1 to <2, 3 to <4, 4 to <5, 5 to <10, ≥10), (e) axillary nodal positivity (0 [referent], 1 to 3, 4 to 10, ≥11), and (f) tumor grade (grade 1 referent). These variables were treated as continuous variables for trend estimates. All statistical tests were two-sided, and *p *values of 0.05 or less were considered significant.

## Results

Within the identified cohort of 155,175 women with known joint ER/PR receptor status, 98,463 cases had ER+/PR+ tumors (63%). Of the remaining women, 19,886 cases had ER+/PR- tumors (13%), 4,896 cases had ER-/PR+ tumors (3%), and 31,930 cases had ER-/PR- tumors (21%). Older women were more likely to be diagnosed with ER+/PR+ tumors, whereas more than one third of women 30 to 39 years old presented with ER-/PR- tumors (Table 1). The proportion of tumors that were ER+/PR+ increased over the study period, whereas the proportions of tumors that were ER+/PR- and ER-/PR- held fairly constant, and the proportion of tumors that were ER-/PR+ declined. In general, for the other characteristics shown in Table 1, ER+/PR+ and ER+/PR- tumors were similar to each other and ER-/PR+ and ER-/PR- tumors were similar to each other. Specifically, compared to women with ER+PR+ and ER+/PR- tumors, those diagnosed with ER-/PR+ and ER-/PR- tumors were somewhat more likely to be younger and African-American, to have larger tumors, more advanced disease stage, and higher tumor grade, and to present with axillary lymph node metastases. In addition, women with ER-/PR+ and ER-/PR- tumors were somewhat less likely to have lobular, ductal/lobular, mucinous, or tubular carcinomas and were somewhat more likely to have inflammatory, comedo, or medullary carcinomas.

The risks of breast cancer-specific mortality were elevated among women with ER+/PR-, ER-/PR+, and ER-/PR- tumors relative to women with ER+/PR+ tumors across all subcategories of age at cancer diagnosis (Table 2). Age was an effect modifier of the relationship between ER/PR status and relative risk of breast cancer mortality (*p *= 0.03). Specifically, HRs for ER-/PR+ and ER-/PR- disease were particularly high among women 65 years of age and older. For each 5-year increase in age, a 5% to 7% elevation in mortality risk was observed within each ER/PR profile (*p *for trend < 0.0001, all profiles) (Table 3). Among the population of women with ER-/PR- tumors relative to women with ER+/PR+ tumors, a higher relative mortality risk was associated with a tumor diagnosed in the most recent years versus a tumor diagnosed in the early 1990s (*p *for interaction < 0.0001). However, decreases in HRs ranging from 4% to 8% per year were observed with increasing calendar year within each ER/PR profile and the magnitude of this reduction was greater among women with ER+ disease than ER- disease. In general, the magnitudes of the HRs associated with each ER/PR profile were comparable across race/ethnicity classifications (*p *for interaction = 0.77).

Elevations in breast cancer mortality risks were observed among women with ER+/PR-, ER-/PR+, and ER-/PR- tumors relative to women with ER+/PR+ tumors across the majority of clinical characteristics examined (Table 2). The magnitudes of these relative risks did not vary appreciably by stage; also, for each increase in tumor stage level, two-fold or greater increases in mortality risks were observed within each ER/PR profile (*p *for trend < 0.0001) (Table 3). Tumor size, lymph node status, histology, and grade appeared to modify the relationship between ER/PR status and relative risk of mortality. For tumor size and grade, the mortality risks associated with ER-/PR+ tumors were particularly high among women whose tumors were either more than 5 cm in size or of high grade. The mortality risks associated with ER-/PR- tumors, relative to ER+/PR+ tumors, were particularly high among women whose tumors were less than 2 cm in size or of low grade (Table 2). In addition, the HRs increased with increasing tumor size, number of axillary lymph node metastases, and disease grade within each ER/PR profile (*p *for trend < 0.0001, all profiles) (Table 3). The magnitude of the associated relative mortality risk depended on ER/PR status. For example, women with ER-/PR- tumors had a 24% increase in the relative risk of breast cancer mortality with each increase in tumor grade level, whereas women with ER+/PR+ tumors had a 62% elevated mortality risk with each increase in tumor grade level. With respect to histology, compared to women with ER+/PR+ tumors, women with ER+/PR- tumors had elevated risks of mortality if their tumor was ductal or lobular, women with ER-/PR+ tumors had elevated risks of mortality if their tumor was ductal or inflammatory, and women with ER-/PR- tumors had elevated risks of mortality across all histologies, except for medullary carcinoma. Due to the sparse number of subjects in each hormonal category and limited or no recorded deaths, data were not shown for mucinous, papillary, and tubular carcinomas.

## Discussion

Previous studies have shown survival advantages among women with hormone receptor-positive tumors relative to women with hormone receptor-negative tumors [6,9,13-16]. A recent study by Grann and coworkers [17] that also used data collected from the SEER program reported that joint ER/PR status was an independent predictor of outcome in a large cohort of women with breast carcinoma. Our study expands on this study, further evaluating the association between ER/PR status and breast cancer-specific mortality within subgroups of women defined by personal characteristics (including race/ethnicity, age at cancer diagnosis, and year of cancer diagnosis) and tumor characteristics (including histology, stage, grade, size, and axillary lymph node metastases). In general, we observed that the higher relative risks of mortality associated with having an ER+/PR-, an ER-/PR+, or an ER-/PR- tumor relative to an ER+/PR+ tumor were consistently present across almost all tumor characteristics. Even among women with poor prognoses, such as those with stage IV disease, multiple positive lymph nodes, or tumors of high grade, differences in the relative risk of mortality by ER/PR status were observed. We also estimated mortality trends by ER/PR status within the study population. Within each ER/PR profile, we document an increase in the relative risk of breast cancer mortality for each 5-year increase in age and for each incremental increase in tumor stage, size, grade, or axillary lymph node metastases. These findings are in agreement with the known correlation between increased breast cancer mortality risk and increasing tumor stage, size, grade, or regional lymph node metastases [18,19]. We observed a decreased mortality trend each year over the study period of 1990 to 2001 which was greatest in magnitude among women with ER+ tumors. This trend may be related to improvements in breast cancer treatments and/or early detection methods resulting in improved patient outcomes.

We did observe some variations in the association between ER/PR status and risk of breast cancer mortality by tumor size, grade, and histology. Relative to ER+/PR+ patients, and within subcategories of tumor size and grade, the highest observed relative mortality risks were among ER-/PR- patients whose tumors were small (0 to 1.9 cm) or of low grade (grade 1 and 2). These elevated risks are likely related to adjuvant treatment standards given that hormonal therapy generally is recommended for women with ER+/PR+ disease, regardless of tumor size. Conversely, adjuvant chemotherapy is not routinely recommended for ER-/PR- patients whose tumors are small and have favorable features (that is, negative lymph nodes, highly differentiated) [20]. Our data also showed that ER-/PR+ patients whose tumors were more than 5 cm in size or of high grade had particularly elevated relative mortality risks (HRs = 2.2 and 2.1, respectively) that were higher than those with ER+/PR- tumors of similar size and grade (HRs = 1.3 and 1.4, respectively), suggesting that ER negativity may have a greater influence on mortality risk than PR negativity among women with these tumor types. Compared to women with ER+/PR+ tumors, those with ER-/PR- tumors had increased risks of mortality across almost all histologic classifications, suggesting that combined ER/PR negativity has implications for relative mortality risk, regardless of tumor histology. The one noted exception was that ER/PR status did not appear to be related to the relative risk of mortality among women with medullary carcinomas. Medullary carcinomas are rare, and although they are typically high-grade, they tend to have well-defined, distinct borders. Their prognosis is more favorable than that of other invasive breast carcinomas, such as ductal carcinoma [21].

Researchers who examined the risk of invasive breast carcinoma diagnosis among women of different races reported that certain ethnicities have elevated risks of presenting with ER-/PR- tumors. African-Americans, Asians, Native Americans, and Hispanic whites were found to have greater risks of presenting with ER-/PR- breast tumors compared to non-Hispanic whites [22-25]. Although it has been shown that women of certain racial/ethnic groups have increased risks of developing hormone receptor-negative tumors, our results show little or no difference in mortality risks within ethnic classes for each ER/PR profile. For example, African-American women whose tumors were ER-/PR+ or ER-/PR- were found to have relative risks of breast cancer mortality similar to non-Hispanic whites with ER-/PR+ or ER-/PR- tumors, respectively.

There are potential study limitations using SEER data. First, ER/PR status, tumor histology, and tumor grade were not assessed centrally since the data recorded by SEER are derived from review of clinical pathology reports. Most importantly, assays and techniques used for ER/PR testing likely varied both across and within laboratories over the course of this study. For example, cutoff points may have been dissimilar in differentiating hormone receptor positivity. High cutoff values may result in tumors being misclassified as ER- [26]. However, assay techniques for ER and PR have improved since their inception nearly 30 years ago and receptor status can be appropriately determined with relative ease [27,28]. In the period to which this study was restricted (1990 to 2001), pathology laboratories in general routinely performed ER/PR testing of breast cancer. Also reassuring is the fact that the proportions of the four joint tumor ER/PR receptor profiles in our study population were comparable to those reported in other studies [13,14].

The exclusion of subjects with no recorded ER/PR data is a second potential limitation of this study. The absence of recorded hormone receptor data has been reported to be associated with age and year of diagnosis, tumor stage, grade, histology, and SEER registries [17,29], and thus the lack of ER/PR data on these cases could bias our results. However, the number of SEER records missing ER/PR data has declined over time and the decline has been shown to be consistent across all age categories [29]. The proportion of records containing ER/PR data for this cohort increased over time, ranging from 67.5% in 1990 to 73% in the years 1994 to 1995 and 80.7% in 2001, results that are consistent with prior reports [17].

Survivorship for SEER registries is tracked through state vital records and the National Death Index (NDI) established by the National Center for Health Statistics (NCHS). Cause-of-death data in relation to death certificate completion or coding are subject to misclassification. However, US death certificates are checked at several levels for completeness before transmission to the NCHS. The NDI is reported to have the highest sensitivity of all major US mortality databases [30]. In addition, a study that evaluated the accuracy of the cause-of-death code found small discrepancy rates (ranging from 4% to 7%) between NDI Plus codes, final study codes, and NCHS nosologists' original codes [31].

The SEER program does not collect data regarding mammography screening program participation within the designated state and metropolitan tumor registries. Mammography screening programs have been shown to improve patient outcomes [32]. The decreased mortality trend we observed each year over the study period, particularly among women with ER+ tumors, may be due in part to screening programs.

A final limitation of this study is that SEER registries do not provide data on the receipt of adjuvant or hormonal therapies following primary surgical and/or radiotherapy interventions. Treating hormone receptor-positive tumors with hormonal therapies has been shown to be a contributing factor in better survival among women with breast cancer [9]. A large proportion of the survival advantage experienced by ER+/PR+ patients compared to ER-/PR- patients may be due to the use of hormonal therapy. Our data indicate that in 1990 to 1992, when the use of hormonal therapy had just begun, ER-/PR- patients had a 1.7-fold greater relative risk of mortality; however, by 1999 to 2001, when hormonal therapy was widely integrated into clinical practice and guidelines for its use were well established, women with ER-/PR- tumors had a 4.9-fold greater relative risk of mortality. In future years, the advent of better chemotherapy treatments for the ER- patient population may result in improved disease-free survival and overall survival. Recent studies have reported that the large survival differences among ER+ patients treated with hormonal therapy versus ER- patients treated with chemotherapy have dwindled and that ER- patients are now deriving a greater benefit from improved chemotherapy regimens with risk reductions as high as 49% [33,34].

## Conclusion

Overall, our findings suggest that the higher risks of mortality in women with ER+/PR-, ER-/PR+, and ER-/PR- tumors, compared to women with ER+/PR+ tumors, are largely independent of the various demographic and clinical tumor characteristics assessed in this study. This indicates that the prognostic utility of ER/PR status is for the most part independent of these other factors. However, the strength of the associations we assessed did vary within subcategories of certain factors. The higher relative mortality risks we identified among ER-/PR- patients with small or low-grade tumors raise the question of whether there may be a beneficial role for adjuvant chemotherapy in this population. The lack of data on adjuvant chemotherapies from SEER limits our ability to make this determination. Other underlying biological factors may account for the observed variations in tumor hormone receptor status and mortality risk, requiring additional research to be conducted.

## Abbreviations

AJCC = American Joint Committee on Cancer; CI = confidence interval; ER = estrogen receptor; HR = hazard ratio; NCHS = National Center for Health Statistics; NCI = National Cancer Institute; NDI = National Death Index; PR = progesterone receptor; SEER = Surveillance, Epidemiology, and End Results.

## Competing interests

The authors declare that they have no competing interests.

## Authors' contributions

LD drafted the study design proposal, extracted and prepared the SEER data for statistical analysis, performed the statistical analysis, and drafted the manuscript. MR participated in the study design and coordination and made substantial contributions to manuscript revisions. CL conceived of the study, participated in the study design and coordination, and made substantial contributions to manuscript revisions. All authors read and approved the final manuscript.

## Figures and Tables

**Table 1:** Selected characteristics among breast cancer cases by hormone receptor status: SEER program, 1990–2001

	ER+PR+			ER+PR-			ER-PR+			ER-PR-		
	(*n *= 98,463)			(*n *= 19,886)			(*n *= 4,896)			(*n *= 31,930)		
				
Characteristics	*n*	Column %	Row %	*n*	Column %	Row %	*n*	Column %	Row %	*n*	Column %	Row %
Age at diagnosis, years												
30–39	4,903	5.0	49.5	885	4.5	8.9	569	11.6	5.7	3,558	11.1	35.9
40–49	18,575	18.9	60.9	2,490	12.5	8.2	1,508	30.8	4.9	7,929	24.8	26.0
50–59	21,807	22.1	61.4	4,464	22.5	12.5	1,161	23.7	3.3	8,094	25.4	22.8
60–69	22,019	22.4	65.9	4,752	23.9	14.2	801	16.4	2.4	5,856	18.3	17.5
70–79	20,667	21.0	68.2	4,662	23.4	15.4	584	11.9	1.9	4,401	13.8	14.5
≥80	10,492	10.6	67.7	2,633	13.2	17.0	273	5.6	1.8	2,092	6.6	13.5
Diagnosis year												
1990–1992	16,500	16.8	61.6	3,751	18.9	14.0	1,111	22.7	4.1	5,435	17.0	20.3
1993–1995	22,087	22.4	61.7	4,514	22.7	12.6	1,485	30.3	4.1	7,728	24.2	21.6
1996–1998	27,734	28.2	64.2	5,239	26.3	12.1	1,367	27.9	3.2	8,884	27.8	20.5
1999–2001	32,142	32.6	65.2	6,382	32.1	12.9	933	19.1	1.9	9,883	31.0	20.0
SEER registry												
Atlanta, GA	5,696	5.8	59.4	1,171	5.9	12.2	381	7.8	4	2,344	7.3	24.4
Connecticut	11,093	11.3	61.4	2,448	12.3	13.6	602	12.3	3.3	3,914	12.3	21.7
Detroit, MI	10,559	10.7	60.4	2,314	11.6	13.2	539	11	3.1	4,063	12.7	23.3
Hawaii	4,149	4.2	67.0	703	3.5	11.3	273	5.6	4.4	1,072	3.4	17.3
Iowa	10,238	10.4	65.2	1,914	9.6	12.2	530	10.8	3.4	3,017	9.4	19.2
Los Angeles, CA	15,586	15.8	61.4	3,399	17.1	13.4	919	18.8	3.6	5,491	17.2	21.6
New Mexico	4,066	4.1	61.8	885	4.5	13.5	193	3.9	2.9	1,435	4.5	21.8
San Francisco-Oakland, CA	13,063	13.3	63.9	2,750	13.8	13.5	652	13.3	3.2	3,957	12.4	19.4
San Jose, CA	5,474	5.5	65.3	1,134	5.7	13.5	228	4.7	2.7	1,548	4.9	18.5
Seattle, WA	14,241	14.5	68	2,517	12.7	12.0	423	8.6	2.0	3,770	11.8	18.0
Utah	4,298	4.4	66.9	651	3.3	10.1	156	3.2	2.5	1,319	4.1	20.5
Race												
Non-Hispanic white	78,805	81.7	65.4	15,716	80.5	13.0	3,515	73.5	2.9	22,532	72.1	18.7
African-American	5,724	5.9	48.0	1,488	7.6	12.5	504	10.5	4.2	4,206	13.5	35.3
Native American	284	0.3	55.0	57	0.3	11.0	23	0.5	4.5	152	0.5	29.5
Asian/Pacific Islander	6,065	6.3	65.1	1,061	5.4	11.4	382	8.0	4.1	1,805	5.8	19.4
Hispanic white	5,585	5.8	57.8	1,203	6.2	12.4	359	7.5	3.7	2,524	8.1	26.1
Other/Unknown	2,000	-	-	361	-	-	113	-	-	711	-	-
Tumor histology												
Ductal	71,199	72.3	62.7	13,981	70.3	12.3	3,641	74.4	3.2	24,722	77.4	21.8
Lobular	8,980	9.1	73.6	2,160	10.9	17.7	283	5.8	2.3	784	2.5	6.4
Ductal/Lobular	7,576	7.7	76.7	1,341	6.7	13.6	233	4.8	2.4	722	2.3	7.3
Inflammatory	543	0.5	37.8	191	1.0	13.3	90	1.8	6.3	611	1.9	42.6
Mucinous	2,927	3.0	80.9	486	2.4	13.5	41	0.8	1.1	164	0.5	4.5
Tubular	1,696	1.7	79	327	1.6	15.2	44	0.9	2.1	79	0.3	3.7
Comedo	1,159	1.2	43.6	318	1.6	12.0	179	3.6	6.7	1,003	3.1	37.7
Medullary	266	0.3	14.7	148	0.8	8.2	97	2.0	5.4	1,292	4.0	71.7
Papillary	582	0.6	79.0	58	0.3	7.9	10	0.2	1.3	87	0.3	11.8
Other	3,535	3.6	49.4	876	4.4	12.2	278	5.7	3.9	2,466	7.7	34.5
Tumor stage												
I	50,172	52.4	69.2	9,232	48	12.7	1,999	42.7	2.8	11,114	36.2	15.3
II	37,335	39.0	60.3	7,606	39.5	12.3	2,068	44.2	3.3	14,878	48.4	24.1
III	5,332	5.5	51.3	1,471	7.6	14.1	385	8.2	3.7	3,210	10.4	30.9
IV	2,943	3.1	52.1	936	4.9	16.6	230	4.9	4.1	1,536	5.0	27.2
Unstaged	2,681	-	-	641	-	-	214	-	-	1,192	-	-
Tumor grade												
1	18,012	21.8	81.1	2,885	17.5	13.0	405	9.9	1.8	914	3.3	4.1
2	40,642	49.3	74.2	7,133	43.4	13.0	1,324	32.5	2.4	5,682	20.6	10.4
3	22,082	26.8	44.4	6,027	36.6	12.1	2,174	53.3	4.4	19,412	70.4	39.1
4	1,774	2.1	45.0	413	2.5	10.5	173	4.3	4.4	1,579	5.7	40.1
Unknown	15,953	-		3,428	-		820	-		4,343	-	
Tumor size (cm)												
0–1.9	56,547	59.9	69.2	10,302	54.6	12.6	2,271	49.5	2.8	12,576	41.8	15.4
2–5	32,977	34.9	58.7	7,135	37.8	12.7	1,891	41.3	3.4	14,147	47.1	25.2
>5	4,949	5.2	48.8	1,432	7.6	14.1	422	9.2	4.2	3,330	11.1	32.9
Unknown	3,990	-	-	1,017	-	-	312	-	-	1,877	-	-
Axillary lymph node status												
Negative	54,948	65.3	64.8	10,428	63.0	12.3	2,622	61.0	3.1	16,749	60.3	19.8
Positive	29,234	34.7	60.9	6,110	37.0	12.7	1,675	39.0	3.5	11,015	39.7	22.9
Unknown	14,281	-	-	3,348	-	-	599	-	-	4,166	-	-
Surgical treatment												
Performed	96,387	98.0	63.7	19,216	96.7	12.7	4,764	97.4	3.2	30,847	96.8	20.4
Not performed	2,012	2.0	52.7	648	3.3	17.0	126	2.6	3.3	1,032	3.2	27
Unknown	64	-	-	22	-	-	6	-	-	51	-	-
Radiation therapy												
Treated	46,859	48.5	65.5	8,694	44.5	12.1	2,048	42.9	2.9	13,922	44.8	19.5
None	49,776	51.5	61.9	10,836	55.5	13.4	2,725	57.1	3.4	17,131	55.2	21.3
Unknown	1,828	-	-	356	-	-	123	-	-	877	-	-

ER, estrogen receptor; PR, progesterone receptor; SEER, Surveillance, Epidemiology, and End Results.

**Table 2:** Breast cancer mortality risk by hormone receptor status among women of different diagnosis age, race/ethnicity, diagnosis year, tumor stage, grade, histology, and axillary lymph node positivity^a^

	ER+/PR+			ER+/PR-				ER-/PR+				ER-/PR-				
	(*n *= 98,463)			(*n *= 19,886)				(*n *= 4,896)				(*n *= 31,930)				
					
Characteristics	No. at risk	No. deaths	HR	No. at risk	No. deaths	HR	95% CI	No. at risk	No. deaths	HR	95% CI	No. at risk	No. deaths	HR	95% CI	*p *value for interaction
All cases^b^	98,463	7,319	1.0	19,886	2,434	1.4	1.3–1.5^c^	4,896	848	1.8	1.6–1.9^c^	31,930	6,300	2.3	2.2–2.4^c^	
Age at diagnosis, years^d^																
<50	16,962	1,042	1.0	2,387	245	1.2	1.1–1.4^c^	1,430	209	1.6	1.4–1.9^c^	8,056	1,323	2.1	1.9–2.3^c^	
50–64	23,467	1,104	1.0	4,745	416	1.5	1.3–1.7^c^	1,072	124	1.5	1.2–1.8^c^	7,651	1,214	2.3	2.1–2.5^c^	
≥65	25,078	1,496	1.0	5,563	540	1.5	1.3–1.7^c^	718	119	2.1	1.8–2.6^c^	5,536	1,020	2.6	2.4–2.8^c^	0.03
Diagnosis year^e^																
1990–1992	8,298	1,285	1.0	1,889	419	1.3	1.2–1.5^c^	597	146	1.6	1.3–1.9^c^	2,850	831	1.7	1.5–1.9^c^	
1993–1995	13,665	1,348	1.0	2,688	428	1.5	1.3–1.7^c^	944	160	1.5	1.2–1.8^c^	4,859	1,234	2.2	2.0–2.4^c^	
1996–1998	19,350	837	1.0	3,493	282	1.5	1.3–1.7^c^	971	121	2.1	1.7–2.6^c^	6,181	1,059	2.8	2.5–3.1^c^	
1999–2001	24,194	172	1.0	4,625	69	1.6	1.2–2.1^c^	708	25	3.6	2.3–5.1^c^	7,353	433	4.9	4.1–6.0^c^	<0.001
Race/Ethnicity^f^																
Non-Hispanic white	53,397	2,831	1.0	10,187	916	1.5	1.3–1.6^c^	2,396	313	1.8	1.6–2.0^c^	15,371	2,462	2.3	2.2–2.5^c^	
African-American	3,536	348	1.0	907	125	1.2	1.0–1.5^c^	293	60	1.7	1.3–2.2^c^	2,734	615	2.2	1.9–2.6^c^	
Native American	196	19	1.0	34	11	1.5	0.5–3.8	12	1	1.7	0.1–16.4	102	25	3.4	1.5–7.3^c^	
Asian/Pacific Islander	4,497	189	1.0	748	63	1.8	1.3–2.4^c^	267	34	1.6	1.1–2.4^c^	1,273	166	2.3	1.8–2.8^c^	
Hispanic white	3,881	255	1.0	819	83	1.3	1.0–1.7^c^	252	44	1.9	1.3–2.6^c^	1,763	289	2.3	1.9–2.8^c^	0.77
Tumor stage^g^																
I	33,821	487	1.0	6,038	146	1.4	1.2–1.7^c^	1,369	51	1.6	1.2–2.1^c^	7,704	391	2.3	2.0–2.7^c^	
II	27,363	2,110	1.0	5,472	674	1.4	1.3–1.6^c^	1,529	248	1.6	1.4–1.9^c^	11,051	2,069	2.3	2.2–2.5^c^	
III	3,410	659	1.0	922	252	1.3	1.2–1.6^c^	245	104	2.1	1.7–2.6^c^	1,940	763	2.3	2.0–2.6^c^	
IV	672	302	1.0	192	102	1.3	1.0–1.6^c^	59	42	1.9	1.3–2.7^c^	357	245	2.3	1.9–2.8^c^	0.45
Tumor size (cm)^h^																
0–1.9	39,418	865	1.0	6,957	243	1.4	1.2–1.6^c^	1,607	91	1.5	1.2–1.9^c^	8,799	715	2.6	2.3–2.9^c^	
2–5	22,579	2,081	1.0	4,781	708	1.5	1.3–1.6^c^	1,331	253	1.8	1.5–2.0^c^	10,214	2,019	2.2	2.1–2.4^c^	
>5	2,718	526	1.0	761	206	1.3	1.1–1.6^c^	217	89	2.2	1.7–2.7^c^	1,730	636	2.2	1.9–2.5^c^	<0.001
Axillary lymph node status^i^																
Negative	42,451	942	1.0	7,924	324	1.6	1.4–1.8^c^	1,898	114	2.0	1.6–2.4^c^	12,541	1,006	2.8	2.6–3.1^c^	
1–3	14,953	1,021	1.0	2,828	324	1.5	1.3–1.7^c^	790	133	1.8	1.5–2.1^c^	4,857	977	2.5	2.3–2.7^c^	
4–10	5,313	861	1.0	1,133	277	1.4	1.2–1.6^c^	332	108	1.7	1.3–2.1^c^	2,268	793	2.4	2.2–2.7^c^	
≥11	2,549	734	1.0	739	249	1.2	1.0–1.4^c^	182	90	1.7	1.3–2.1^c^	1,386	692	2.2	1.9–2.4^c^	0.08
Tumor histology^j^																
Ductal	52,178	2,919	1.0	9,978	994	1.5	1.4–1.6^c^	2,711	390	1.8	1.6–2.0^c^	18,542	3,040	2.3	2.2–2.5^c^	
Lobular	3,975	186	1.0	938	64	1.4	1.0–1.9^c^	123	3	0.4	0.1–1.4	325	47	1.9	1.4–2.7^c^	
Ductal/Lobular	5,472	254	1.0	929	59	1.3	0.9–1.7	148	13	1.1	0.6–2.0	511	83	2.7	2.1–3.5^c^	
Inflammatory	241	52	1.0	77	22	1.4	0.8–2.3	39	19	3.4	1.9–6.1^c^	258	123	3.5	2.4–5.0^c^	
Comedo	652	91	1.0	187	19	0.6	0.4–1.1	91	16	1.2	0.7–2.1	556	109	1.6	1.2–2.1^c^	
Medullary	120	14	1.0	73	6	0.7	0.2–2.2	48	4	0.9	0.2–3.0	716	54	0.7	0.4–1.4	<0.001
Tumor grade^k^																
1	13,854	156	1.0	2,141	40	1.6	1.1–2.3^c^	310	6	1.0	0.4–2.5	661	32	2.6	1.7–3.9^c^	
2	32,661	1,349	1.0	5,555	374	1.5	1.3–2.1^c^	1,074	95	1.7	1.3–2.1^c^	4,312	575	3.1	2.8–3.4^c^	
3	17,352	1,873	1.0	4,613	710	1.3	1.2–1.5^c^	1,694	319	1.7	1.5–1.9^c^	14,967	2,635	2.1	1.9–2.2^c^	
4	1,399	180	1.0	315	50	1.4	1.0–2.0^c^	124	25	2.1	1.4–3.3^c^	1,112	226	2.3	1.8–2.8^c^	<0.001

^a^The reference ER/PR profile for all analyses was ER+/PR+. ^b^HRs adjusted for age and year at diagnosis, race, SEER registry, tumor histology, grade, stage, surgical and radiation treatment, and lymph node status (positive/negative). ^c^*p *< 0.05. ^d^HRs adjusted for year at diagnosis, race, SEER registry, tumor histology, grade, stage, surgical and radiation treatment, and lymph node status (positive/negative). ^e^HRs adjusted for age at diagnosis, race, SEER registry, tumor histology, grade, stage, surgical and radiation treatment, and lymph node status (positive/negative). ^f^HRs adjusted for age and year at diagnosis, SEER registry, tumor histology, grade, stage, surgical and radiation treatment, and lymph node status (positive/negative). Race unknown, *n *= 3,185. ^g^HRs adjusted for age and year at diagnosis, race, SEER registry, tumor histology, grade, lymph node status (categorical), and surgical and radiation treatment. Stage unknown, *n *= 4,728. ^h^HRs adjusted for age and year at diagnosis, race, SEER registry, tumor histology, grade, lymph node status (categorical), and surgical and radiation treatment. Size unknown, *n *= 7,196. ^i^HRs adjusted for age and year at diagnosis, race, SEER registry, tumor histology, stage, grade, and surgical and radiation treatment. Nodal status unknown, *n *= 22,394. ^j^HRs adjusted for age and year at diagnosis, race, SEER registry, tumor grade, stage, lymph node status (categorical), and surgical and radiation treatment. Other/unknown histology, *n *= 7,155. ^k^HRs adjusted for age and year at diagnosis, race, SEER registry, tumor histology, stage, lymph node status (categorical), and surgical and radiation treatment. Grade unknown, *n *= 24,544. CI, confidence interval; ER, estrogen receptor; HR, hazard ratio; PR, progesterone receptor; SEER, Surveillance, Epidemiology, and End Results.

**Table 3:** Trends in hazard ratios for breast cancer mortality associated with hormone receptor status and different demographic and clinical characteristics

	ER+/PR+		ER+/PR-		ER-/PR+		ER-/PR-	
				
Characteristics	HR	95% CI	HR	95% CI	HR	95% CI	HR	95% CI
Age at diagnosis, per 5 years^a^	1.05	1.04–1.06	1.07	1.04–1.09	1.07	1.03–1.10	1.06	1.05–1.07
Diagnosis year, per year^b^	0.93	0.92–0.94	0.92	0.90–0.94	0.95	0.91–0.98	0.96	0.95–0.97
Tumor stage^c^	2.33	2.20–2.46	2.29	2.09–2.51	2.55	2.19–2.96	2.06	1.94–2.18
Tumor size (cm)^d^	1.27	1.24–1.30	1.29	1.24–1.34	1.33	1.25–1.41	1.22	1.20–1.25
Axillary lymph node status^e^	1.71	1.65–1.78	1.55	1.46–1.66	1.61	1.44–1.80	1.64	1.58–1.70
Tumor grade^f^	1.62	1.55–1.70	1.49	1.36–1.63	1.72	1.46–2.03	1.24	1.16–1.32

^a^Five-year categories. Reference = 30 to <35 years. HRs adjusted for year at diagnosis, race, SEER registry, tumor histology, grade, stage, surgical and radiation treatment, and lymph node status (positive/negative). ^b^Reference = 1990. HRs adjusted for age at diagnosis, race, SEER registry, tumor histology, grade, stage, surgical and radiation treatment, and lymph node status (positive/negative). ^c^Reference = stage 1. HRs adjusted for age and year at diagnosis, race, SEER registry, tumor histology, grade, lymph node status, and surgical and radiation treatment. Stage unknown, *n *= 4,728. ^d^Categories = 0 to <1, 1 to <2, 2 to <3, 3 to <4, 4 to <5, 5 to <10, ≥10 cm. Reference = 0 to <1 cm. HRs adjusted for age and year at diagnosis, race, SEER registry, tumor histology, grade, lymph node status, and surgical and radiation treatment. Size unknown, *n *= 7,196. ^e^Categories = 0, 1 to 3, 4 to 10, ≥11. Reference = 0. HRs adjusted for age and year at diagnosis, race, SEER registry, tumor histology, stage, grade, and surgical and radiation treatment. Nodal status unknown, *n *= 22,394. ^f^Reference = grade 1. HRs adjusted for age and year at diagnosis, race, SEER registry, tumor histology, stage, lymph node status, and surgical and radiation treatment. Grade unknown, *n *= 24,544. CI, confidence interval; ER, estrogen receptor; HR, hazard ratio; PR, progesterone receptor; SEER, Surveillance, Epidemiology, and End Results.

## References

[B1] Fisher B, Redmond C, Fisher ER, Caplan R (1988). Relative worth of estrogen or progesterone receptor and pathologic characteristics of differentiation as indicators of prognosis in node negative breast cancer patients: findings from National Surgical Adjuvant Breast and Bowel Project Protocol B-06. J Clin Oncol.

[B2] Parl FF, Schmidt BP, Dupont WD, Wagner RK (1984). Prognostic significance of estrogen receptor status in breast cancer in relation to tumor stage, axillary node metastasis, and histopathologic grading. Cancer.

[B3] Crowe JP, Gordon NH, Hubay CA, Shenk RR, Zollinger RM, Brumberg DJ, McGuire WL, Shuck JM (1991). Estrogen receptor determination and long term survival of patients with carcinoma of the breast. Surg Gynecol Obstet.

[B4] Aaltomaa S, Lipponen P, Eskelinen M, Kosma VM, Marin S, Alhava E, Syrjanen K (1991). Hormone receptors as prognostic factors in female breast cancer. Ann Med.

[B5] Lethaby AE, Mason BH, Harvey VJ, Holdaway IM (1996). Survival of women with node negative breast cancer in the Auckland region. N Z Med J.

[B6] Anderson WF, Chu KC, Chatterjee N, Brawley O, Brinton LA (2001). Tumor variants by hormone receptor expression in white patients with node-negative breast cancer from the surveillance, epidemiology, and end results database. J Clin Oncol.

[B7] Smith RE, Good BC (2003). Chemoprevention of breast cancer and the trials of the National Surgical Adjuvant Breast and Bowel Project and others. Endocr Relat Cancer.

[B8] Goldhirsch A, Wood WC, Gelber RD, Coates AS, Thurlimann B, Senn HJ (2003). Meeting highlights: updated international expert consensus on the primary therapy of early breast cancer. J Clin Oncol.

[B9] Fisher B, Jeong JH, Bryant J, Anderson S, Dignam J, Fisher ER, Wolmark N (2004). Treatment of lymph-node-negative, oestrogen-receptor-positive breast cancer: long-term findings from National Surgical Adjuvant Breast and Bowel Project randomised clinical trials. Lancet.

[B10] Surveillance, Epidemiology, and End Results (SEER) Program Public-Use Data (1973–2001), National Cancer Institute, Division of Cancer Control and Population Sciences, Surveillance Research Program, Cancer Statistics Branch, released April based on the November 2003 submission. http://www.seer.cancer.gov.

[B11] Cox DR (1972). Regression models and life tables (with discussion). J R Stat Soc Ser B.

[B12] Kalbfleisch JD, Prentice RL (2002). The Statistical Analysis of Failure Time Data.

[B13] Pichon MF, Broet P, Magdelenat H, Delarue JC, Spyratos F, Basuyau JP, Saez S, Rallet A, Courriere P, Millon R (1996). Prognostic value of steroid receptors after long-term follow-up of 2257 operable breast cancers. Br J Cancer.

[B14] Bernoux A, de Cremoux P, Laine-Bidron C, Martin EC, Asselain B, Magdelenat H (1998). Estrogen receptor negative and progesterone receptor positive primary breast cancer: pathological characteristics and clinical outcome. Institut Curie Breast Cancer Study Group. Breast Cancer Res Treat.

[B15] Costa SD, Lange S, Klinga K, Merkle E, Kaufmann M (2002). Factors influencing the prognostic role of oestrogen and progesterone receptor levels in breast cancer – results of the analysis of 670 patients with 11 years of follow-up. Eur J Cancer.

[B16] Bardou VJ, Arpino G, Elledge RM, Osborne CK, Clark GM (2003). Progesterone receptor status significantly improves outcome prediction over estrogen receptor status alone for adjuvant endocrine therapy in two large breast cancer databases. J Clin Oncol.

[B17] Grann VR, Troxel AB, Zojwalla NJ, Jacobson JS, Hershman D, Neugut AI (2005). Hormone receptor status and survival in a population-based cohort of patients with breast carcinoma. Cancer.

[B18] Donegan WL (1992). Prognostic factors. Stage and receptor status in breast cancer. Cancer.

[B19] Cianfrocca M, Goldstein LJ (2004). Prognostic and predictive factors in early-stage breast cancer. Oncologist.

[B20] National Cancer Comprehensive Network (NCCN) Clinical Practice Guidelines in Oncology, Breast Cancer, V.I.2007. http://www.nccn.org/professionals/physician_gls/default.asp.

[B21] Li CI, Uribe DJ, Daling JR (2005). Clinical characteristics of different histologic types of breast cancer. Br J Cancer.

[B22] Gapstur SM, Dupuis J, Gann P, Collila S, Winchester DP (1996). Hormone receptor status of breast tumors in black, Hispanic, and non-Hispanic white women. An analysis of 13,239 cases. Cancer.

[B23] Li CI, Malone KE, Daling JR (2002). Differences in breast cancer hormone receptor status and histology by race and ethnicity among women 50 years of age and older. Cancer Epidemiol Biomarkers Prev.

[B24] Pegoraro RJ, Karnan V, Nirmul D, Joubert SM (1986). Estrogen and progesterone receptors in breast cancer among women of different racial groups. Cancer Res.

[B25] Elledge RM, Clark GM, Chamness GC, Osborne CK (1994). Tumor biologic factors and breast cancer prognosis among white, Hispanic, and black women in the United States. J Natl Cancer Inst.

[B26] Osborne CK (1998). Steroid hormone receptors in breast cancer management. Breast Cancer Res Treat.

[B27] Allred DC, Bustamante MA, Daniel CO, Gaskill HV, Cruz AB (1990). Immunocytochemical analysis of estrogen receptors in human breast carcinomas. Evaluation of 130 cases and review of the literature regarding concordance with biochemical assay and clinical relevance. Arch Surg.

[B28] Harvey JM, Clark GM, Osborne CK, Allred DC (1999). Estrogen receptor status by immunohistochemistry is superior to the ligand-binding assay for predicting response to adjuvant endocrine therapy in breast cancer. J Clin Oncol.

[B29] Li CI, Daling JR, Malone KE (2003). Incidence of invasive breast cancer by hormone receptor status from 1992 to 1998. J Clin Oncol.

[B30] Cowper DC, Kubal JD, Maynard C, Hynes DM (2002). A primer and comparative review of major US mortality databases. Ann Epidemiol.

[B31] Sathiakumar N, Delzell E, Abdalla O (1998). Using the National Death Index to obtain underlying cause of death codes. J Occup Environ Med.

[B32] (2002). IARC Handbooks of Cancer Prevention Breast Cancer Screening.

[B33] International Breast Cancer Study Group (2002). Endocrine responsiveness and tailoring adjuvant therapy for postmenopausal lymph node-negative breast cancer: a randomized trial. J Natl Cancer Inst.

[B34] Berry DA, Cirrincione C, Henderson IC, Citron ML, Budman DR, Goldstein LJ, Martino S, Perez EA, Muss HB, Norton L (2006). Estrogen-receptor status and outcomes of modern chemotherapy for patients with node-positive breast cancer. JAMA.

